# Utility of hydroxychloroquine laboratory monitoring in dermatologic and rheumatologic patients

**DOI:** 10.1007/s00403-024-02876-7

**Published:** 2024-05-22

**Authors:** Maxwell Green, Laura Williams, Erin Boh, Drew Kuraitis

**Affiliations:** 1 Tulane University School of Medicine, New Orleans, LA USA; 2 Department of Dermatology, Tulane University School of Medicine, New Orleans, LA USA; 3 Department of Dermatology, Roswell Park Cancer Center, 665 Elm St, Buffalo, NY 14263 USA

**Keywords:** Hydroxychloroquine, Laboratory monitoring, Monitoring studies, Medication side effects, Comprehensive metabolic panel, Complete blood count, General dermatology, Rheumatology

## Abstract

Hydroxychloroquine (HCQ) is an immunomodulator used in dermatology and rheumatology. Side effects may be observed on routine monitoring studies before they become clinically apparent. The goal of this retrospective chart review was to assess laboratory abnormalities in dermatologic and rheumatologic patients taking HCQ. Medical records of patients prescribed HCQ were retrospectively reviewed. Demographics, reported side effects, and parameters on baseline and follow-up complete blood count (CBC) and comprehensive metabolic panel (CMP) were recorded and graded. Laboratory abnormalities were considered severe if they were grade 3 or greater according to *Common Terminology Criteria for Adverse Events v3.0* and persistent if they continued beyond subsequent laboratory testing. Of 646 eligible charts, 289 had monitoring studies for review. There were 35 severe (grade 3 or 4, 35/289; 12%) adverse events that developed, as noted on CBC or CMP. Of these 35 severe adverse events, 25 self-corrected on subsequent testing, and 10 (10/289, 3%) across 9 patients were persistent, including glomerular filtration rate, alanine transferase, alkaline phosphatase, glucose, hemoglobin and lymphopenia abnormalities. Of these 10 abnormalities, 7/10 (70%) were unlikely due to hydroxychloroquine use according to the calculated Naranjo score for each patient. Severe laboratory abnormalities while taking hydroxychloroquine are rare, even in a population with a high rate of comorbidities. Among the abnormalities observed, the majority of them (70%) were likely due to disease progression or a medication other than hydroxychloroquine. CBC and CMP monitoring for the reason of observing abnormalities while on HCQ should be at the discretion of the prescribing physician.

## Introduction

Hydroxychloroquine (HCQ) is an oral immunomodulatory treatment for a wide variety of rheumatologic and dermatologic inflammatory conditions. Although generally well-tolerated, there are few important side effects associated with hydroxychloroquine use, such as ocular toxicity, hepatotoxicity, mucocutaneous eruptions, gastrointestinal upset, and rare cases of bone marrow toxicity [1]. These side effects may present with symptomatic involvement, or they may be detected before becoming clinically apparent using routine lab monitoring studies. Comprehensive metabolic panel (CMP) and complete blood count (CBC) monitoring remains commonplace among physicians prescribing hydroxychloroquine to monitor for evidence of anemia, agranulocytosis, or hepatotoxicity; however, current guidelines regarding laboratory monitoring while on HCQ remain poorly defined within dermatology [2]. A recent survey of practicing dermatologists regarding HCQ lab monitoring noted that 18% do not obtain any baseline lab monitoring, up to 23% obtain laboratory studies every 2–4 months while on HCQ and 22% do not check CBC or CMP at all while on HCQ [3].

Guidelines from the American College of Rheumatology regarding HCQ use for rheumatoid arthritis recommend baseline CBC, liver transaminases and creatinine before starting hydroxychloroquine, without specific recommendations for routine follow-up lab monitoring [4]. A recent analysis of HCQ use in dermatology suggested that periodic CBCs may be checked to evaluate for HCQ-induced blood dyscrasias, as well as intermittent CMP checking to evaluate for hepatotoxicity [5]. Although hepatotoxicity has rarely been reported, an important exception exists, as hydroxychloroquine has been associated with acute liver injury during treatment of porphyria cutanea tarda [6]. Formal guidelines for lab monitoring in dermatology do not exist, and literature regarding HCQ safety and laboratory abnormalities is sparse. Thus, the goal of this retrospective chart review was to evaluate the frequency of CBC and CMP abnormalities in patients taking hydroxychloroquine to determine the utility of laboratory monitoring.

## Methods

Patient data were extracted from Tulane University clinic electronic health records (EHR). Patients over 18 years of age prescribed hydroxychloroquine from 2012–2021 were included, with those prescribed the drug for COVID-19 excluded from the study. Medication start dates were determined from physician visit encounters and any end dates were recorded when discontinuation was noted. The study 2021–907 was approved by Tulane University Institutional Review Board.

Subject demographic information from EHR was recorded, including age, gender, concomitant medications and medical condition for which hydroxychloroquine was prescribed. Additionally, any side effects experienced by the patient as reported in EMR notes were recorded. Laboratory values were only recorded for patients who had baseline CMP and/or CBC available through Tulane University clinics. Labs were considered baseline if they were drawn within 90 days prior to the start of hydroxychloroquine or within one month of initiation. Any labs drawn after this period were considered monitoring labs. Number of patient comorbidities, concomitant medications, and number of tests reviewed are reported as mean ± standard error.

For patients with this available laboratory data, CBC and CMP parameters were recorded through the course of the patients’ treatment regimen; absolute white blood cell count (WBC), hemoglobin, absolute neutrophil count, absolute lymphocyte count, platelet count, glucose, glomerular filtration rate (GFR), serum creatinine, bilirubin, aspartate aminotransferase (AST), alkaline phosphatase (AP), and alanine aminotransferase levels (ALT). Any lab abnormality noted either on baseline labs or monitoring labs was graded using the *Common Terminology Criteria for Adverse Events v3.0.* [7]. Per this grading system, grade 1 abnormalities are classified as mild and grade 2 abnormalities classified as moderate, indicating that no or a mild, noninvasive intervention would be indicated to correct the abnormality. Grade 3 and 4 abnormalities are classified as severe and life-threatening respectively, and hospitalization and/or immediate intervention would be indicated. For our study, the most severe monitoring abnormality was used across all monitoring labs for each patient. Descriptive statistics for demographic data and discrete data including raw numbers and percentages for lab abnormalities were calculated and reported for results.

## Results

A total of 646 patient charts were screened. Most patients were women (82%; 530/646) with an average age of 56.3 years. Included patients were taking an average of 7.4 medications in addition to hydroxychloroquine (standard error = 0.39) and had an average of 6.8 concurrent comorbidities (standard error = 0.40). Rheumatologic conditions were treated more frequently than dermatologic conditions, specifically rheumatoid arthritis (RA) (n = 133/646, 20.3%), inflammatory arthritis (n = 90/646, 13.9%), and systemic lupus erythematous (SLE) (n = 88/646, 13.6%). The most common dermatologic conditions treated were lichen planus (n = 33/646, 5.1%), dermatomyositis (n = 21/646, 3.3%), discoid lupus (n = 15/646, 2.3%), granuloma annulare (n = 8/646, 1.2%), lichen planopilaris (n = 7/646, 1.1%), and morphea (n = 7/646, 1.1%). 76 patients reported symptomatic side effects, most commonly cutaneous reactions (n = 29/646, 4.9%), gastrointestinal complaints (n = 18/646, 2.8%), and ophthalmologic concerns (n = 16/646, 2.5%). The most common ophthalmologic concerns included eye irritation/burning (n = 9/16, 56.3%), blurred vision (n = 3/16, 18.8%), and vision changes (n = 3/16, 18.8%). Overall, 12 patients discontinued due to side effects: 4 patients self-discontinued the medication due to ophthalmologic concerns, 2 patients were discontinued by ophthalmology, and 6 patients self-discontinued the medication due to cutaneous or GI side effects. No patients reported side effects of bleeding or bruising. These results are summarized in Table 1.

**Table 1:** Demographic Information, Indication for Hydroxychloroquine, and Reported Side Effects of Patients Prescribed Hydroxychloroquine (N = 646)

Variable	Result	Sample Size
Age	Mean = 56.3 years	N = 646
Sex	82% female	N = 530/646
Condition		N = 646
Rheumatoid arthritis	20.6%	N = 133/646
Inflammatory polyarthritis	13.9%	N = 90/646
Systemic lupus erythematosus	13.6%	N = 88/646
Sarcoidosis	9.1%	N = 59/646
Sjogren’s	2.9%	N = 19/646
Lichen Planus	5.1%	N = 33/646
Dermatomyositis	3.3%	N = 21/646
Discoid lupus	2.3%	N = 15/646
Granuloma annulare	1.2%	N = 8/646
Lichen planopilaris	1.1%	N = 7/646
Morphea	1.1%	N = 7/646
Other	25..7%	N = 166/646
Patient-reported side effects	11.8% of patients	N = 76/646
Cutaneous reaction	4.9%	N = 29/646
Gastrointestinal concern	2.8%	N = 18/646
Ophthalmologic concern	2.5%	N = 16/646
Other	2.1%	N = 13/646

Of the 646 patient charts screened, 289 patient charts had baseline and monitoring labs for both CBC and CMP review, meeting inclusion criteria for laboratory monitoring analysis. Patients had an average of 4.23 ± 0.3 monitoring CMPs and 4.63 ± 0.3 monitoring CBCs over an average of 42.5 monitoring months (standard error = 2.1 years). The most common mild and moderate abnormalities were GFR, hemoglobin, and glucose (Table 2). A total of 35 (35/289; 12%) severe adverse events, representing a grade 3 or greater abnormality, developed during lab monitoring (n = number of abnormalities that self-corrected on subsequent lab test/all observed grade 3 or 4 abnormalities): 1 × alanine transferase (n = 0/1), 2 × aspartate transaminase (n = 2/2), 1 × alkaline phosphatase (n = 0/1), 8 × glucose (n = 6/8), 3 × glomerular filtration rates (n = 0/3), 1 × creatinine (n = 1/1), 1 × leukopenia (n = 1/1), 5 × hemoglobin (n = 4/5), 5 × neutropenia (n = 5/5), and 8 × lymphopenia (n = 6/8) There were no bilirubin or platelet severe abnormalities that developed. Across all laboratory values, only 10 of 35 (10/289; 3%) grade 3 or 4 abnormalities persisted beyond subsequent testing, with the remaining 25 abnormalities correcting without intervention by the next CMP or CBC. The 10 persisting severe abnormalities, across 9 patients, included 1 alanine transferase, 1 alkaline phosphatase, 2 glucose, 3 GFR, 1 hemoglobin, and 2 lymphocyte abnormalities. Of the 9 patients with persistent severe lab abnormalities, two were discontinued from the medication. Both of these patients had severe GFR abnormalities, with one self-discontinuing the medication and the other advising discontinuation by an outside physician. The medication lists and any changes to medications prior to the onset of grade 3 or greater abnormalities for each patient are listed in Table 3. Additionally, a Naranjo Adverse Drug Reaction Probability Scale Score was calculated for hydroxychloroquine in each of the 9 patients. The majority of patients (n = 6/9, 66.7%) representing the majority of laboratory abnormalities (n = 7/10, 70.0%) received a score of 0, meaning the laboratory abnormality was likely due to something other than hydroxychloroquine [8].

**Table 2:** Frequency of Lab Abnormalities in Patients with Baseline and Monitoring Labs while taking Hydroxychloroquine (N = 289)

Test	Grade 1 or 2 abnormalities	Grade 3 abnormalities	Percentage of severe adverse events that developed
ALT			
Baseline	11	0	
Monitoring	27	1	0.35% (N = 1/289)
AST			
Baseline	16	0	
Monitoring	37	2	0.69% (N = 2/289)
AP			
Baseline	28	0	
Monitoring	55	1	0.35% (N = 1/289)
Glucose			
Baseline	70	1	
Monitoring	97	7	2.08% (N = 6/289)
GFR			
Baseline	141	6	
Monitoring	162	9	1.04% (N = 3/289)
Creatinine			
Baseline	40	2	
Monitoring	64	3	0.35% (N = 1/289)
Total bilirubin			
Baseline	7	0	
Monitoring	20	0	0%
White blood cells			
Baseline	33	0	
Monitoring	66	1	0.35% (N = 1/289)
Hemoglobin			
Baseline	72	1	
Monitoring	102	6	1.73% (N = 5/289)
Absolute neutrophils			
Baseline	10	1	
Monitoring	31	6	1.73% (N = 5/289)
Absolute lymphocytes			
Baseline	16	2	
Monitoring	50	10	2.77% (N = 8/289)
Platelets			
Baseline	5	0	
Monitoring	19	0	0%

**Table 3:** Characteristics and medication lists for patients with persistent grade 3 or greater laboratory abnormalities (n = 9 patients with 10 abnormalities)

	Type of abnormality	Condition treated	Concurrent meds	Change in meds before abnormality?	Naranjo score for Hydroxychloroquine
Patient 1	Glucose	Sarcoidosis	Glimepiride, Sertraline, Losartan, Ranitidine, Metformin	No	0, doubtful
Patient 2	Lymphocyte	Livedo reticularis	Amlodipine, Aspirin	No	2, possible
Patient 3	Hemoglobin	Systemic lupus erythematous	Mycophenolate, Amlodipine, Spironolactone, Losartan, Chlorthalidone	Mycophenolate increased to 500 mg BID at appointment 1 month prior	1, possible
Patient 4 (2 abnormalities)	Alanine transferase, Alkaline phosphatase	Rheumatoid arthritis	Hydroxyzine, Sertraline, Fexofenadine, Ursodiol, Rifampin	No	0, doubtful
Patient 5	Lymphocyte	Systemic lupus erythematous	Omeprazole, Mycophenolate Prednisone, Amlodipine, Lisinopril	Prednisone started 2–3 weeks prior	1, possible
Patient 6	Glucose	Rheumatoid arthritis	Insulin lispro, Atorvastatin, Candesartan, Metformin, Tamsulosin, Methotrexate, Metoprolol	Atorvastatin increased to 40 mg 8 weeks prior	0, doubtful
Patient 7	GFR	Cryoglobulinemia	Aspirin, Gabapentin, Mycophenolate, Hydrochlorothiazide	No	0, doubtful
Patient 8	GFR	Systemic lupus erythematous	Furosemide, Warfarin, Benazepril, Levothyroxine, Gabapentin, Atorvastatin	Furosemide dose doubled 2 months prior	0, doubtful
Patient 9	GFR	Granuloma annulare	Pentoxifylline, remainder of meds unknown	Unknown	0, doubtful

LiverTox: Clinical and Research Information on Drug-Induced Liver Injury [Internet]. Bethesda (MD): National Institute of Diabetes and Digestive and Kidney Diseases; 2012–. Adverse Drug Reaction Probability Scale (Naranjo) in Drug Induced Liver Injury. 2019 May 4. PMID: 31689026

*GFR* glomerular filtration rate

## Discussion

HCQ has been widely used in dermatology and rheumatology for years. It is thought to be generally safe, but there has not been much critical analysis of its safety, particularly in sicker patient populations. In this study, a total of 35 severe lab abnormalities developed, with most self-resolving and only 10 abnormalities persisting as severe on subsequent testing. Although the development of these abnormalities may have been linked to the use of hydroxychloroquine, they may also have been caused by existing comorbidities in the study population, with the average included patient having 6.8 comorbidities at baseline, 147 of 289 patients having baseline GFR abnormalities and 73 of 289 patients having at least mild anemia. Additionally, no patients were excluded due to the use of other medications, and patients on average were concurrently taking 7.4 medications, some of which may have been hepatotoxic, nephrotoxic or have adverse events on bone marrow-derived cells. Lastly, it is not standard-of-care to monitor labs on patients deemed otherwise healthy while on HCQ, and there is some physician judgement as to whether labs should be monitored while on HCQ therapy, likely influenced by comorbidities and concurrent medications. Our study population included a high rate of complex patients, and due to lack of available subjects who were deemed lower risk and not monitored over time, also did not include patients who would be considered less risky, thus limiting application of our results to the general population. Thus, although 35 severe lab events developed in patients, only 10 persisted beyond subsequent lab testing. Of note, there were 5 cases of severe neutropenia that self-resolved on subsequent testing. These 5 patients were all Black women with SLE, so this finding may be related to Duffy-null Associated Neutrophil Count (DANC), a common finding in patients of African descent with no increased risk for infection [9]. Additionally, SLE can cause either primary or secondary neutropenia from circulating antineutrophil antibodies, which may be another explanation for the lab abnormaltiies seen in this cohort of patients [10].

After applying the Naranjo scoring system for adverse effects due to hydroxychloroquine, 7/10 severe abnormalities across 9 patients were “doubtfully” linked to the use of hydroxychloroquine. Thus, the true rate of severe lab abnormality development in a patient population was likely oversampled in these higher risk patients. Additional study limitations include potential inaccuracies in maintaining active comorbidities and concomitant medications in the EMR.

Overall, the rate of persistently severe lab abnormalities developing in patients remained low (n = 9/289, 3.1%). Of the 9 patients with persistent lab abnormalities, most had comorbidities that may have contributed. All three patients that developed severe GFR abnormalities had Stage 3 CKD at baseline. In addition, the severe ALT and alkaline phosphatase abnormalities that did not resolve developed in a patient with primary sclerosing cholangitis. Next, the patient with unresolving severe anemia had worsening lupus nephritis. Similarly, one patient with lymphopenia had worsening nephrotic syndrome secondary to lupus. For the two patients with unresolving glucose abnormalities, one had CKD and poorly controlled type 2 diabetes, and both patients showed improving glucose levels on subsequent lab testing over the next year. Finally, another patient with severe lymphopenia that persisted was discontinued from hydroxychloroquine, and their numbers improved over the next 6 months. From these patients with grade 3 or greater abnormalities, many had contributory comorbidities at baseline. Physicians should thus use their discretion for further CMP/CBC monitoring for sicker populations patients prescribed hydroxychloroquine. Reported side effects among patients in the study occurred at lower rates compared to rates in the literature. It has been reported that gastrointestinal complaints can occur in almost 1/3 of patients when beginning the medication; in this study, rates may have been lower as GI concerns were not ascertained directly from the patient but instead taken from the electronic medical record. Rates of cutaneous and ophthalmologic side effects, though, were similar to other reports in the literature [11]. Furthermore, one of the most concerning potential side effects associated with hydroxychloroquine is retinopathy, most recently reported to occur in around 1.6% of patients taking hydroxychloroquine for greater than 5 years [12]. There were no reports of retinopathy in our study; however, most patients were not on therapy beyond 5 years, and thus, evaluating prevalence of retinopathy was beyond the scope of this study. However, of the 16 patients who experienced ocular side effects, 4 were self-discontinued from the medication and 2 were discontinued by ophthalmology. This may represent the heightened concern retinopathy development by physicians and patients prescribed HCQ.

Some dermatologists practice vigilant patient surveillance while on HCQ, with routine lab monitoring as frequent as every 2–4 months [3]. HCQ’s parent medication, chloroquine, has a less favorable side effect profile and is likely less commonly used because of this [13]. We would expect that those who are familiar with chloroquine toxicity and lab monitoring may be more likely to perform judicious laboratory evaluations on patients who are taking HCQ. Although infrequent, the most common abnormalities that developed were hematologic in nature, suggesting the utility of CBC monitoring for high-risk patients. Additionally, although rare, reports of aplastic crises occurring after several months of hydroxychloroquine use have been shown in the literature [14]. Thus, based on the small chance of an aplastic crisis developing and the most common abnormalities being hematologic within our study, CBC monitoring may be beneficial and could be performed at the discretion of the physician. Given the rarity of severe CMP abnormalities that do not self-correct on follow-up testing, CMP monitoring for healthy patients may not be beneficial. CMP monitoring should be considered in patients with preexisting conditions that may warrant further oversight, such as liver or kidney disease. Future cohort studies should investigate which patient groups are at higher risk for lab abnormalities and whether these abnormalities are of clinical significance, with hopes of informing clearer guidance on laboratory monitoring.
